# A Sticky Situation: Rare Case of Infected Thrombus in the Superior Vena Cava

**DOI:** 10.51894/001c.144203

**Published:** 2025-09-30

**Authors:** Jeff Justin Aguilar

**Affiliations:** 1 Internal Medicine Detroit Medical Center-Sinai Grace Hospital

## 57

### INTRODUCTION

Catheter-associated thrombus formation is a well-recognized complication, but infected thrombi within the superior vena cava (SVC) are rarely reported.

### CASE DESCRIPTION

A 54-year-old male with hypertension, diabetes, and end-stage renal disease on hemodialysis presented with a non-functioning Permcath. He was found to have atrial fibrillation with ST elevation, and emergent catheterization revealed 100% occlusion of both the left anterior descending and right coronary arteries. Revascularization was unsuccessful, and he was stabilized with an intra-aortic balloon pump and norepinephrine.

After his Permcath site showed drainage, blood cultures grew methicillin-sensitive Staphylococcus aureus (MSSA), prompting catheter removal and IV antibiotics. Transthoracic echocardiogram (TTE) on day 2 showed no thrombi or vegetations. His ICU course deteriorated with sepsis and Proteus bacteremia secondary to acute cholecystitis, requiring percutaneous cholecystostomy. A repeat TTE on day 6 remained unchanged, but a transesophageal echocardiogram (TEE) on day 8 identified mobile echodensities in the SVC, suggestive of vegetations. On day 9, he developed septic and cardiogenic shock, requiring intubation and vasopressor support. He succumbed to multi-organ failure on day 11 after transitioning to comfort care.

### DISCUSSION

This case highlights the rare occurrence of infected SVC thrombi in MSSA bacteremia, detected only by TEE. It underscores the importance of early diagnostic imaging, particularly TEE, in catheter-related bloodstream infections with persistent bacteremia. Current guidelines favor TEE over TTE for detecting intravascular complications in high-risk patients, emphasizing the need for early recognition to reduce morbidity and mortality.

